# Inflammation early in life is a vulnerability factor for emotional behavior at adolescence and for lipopolysaccharide-induced spatial memory and neurogenesis alteration at adulthood

**DOI:** 10.1186/s12974-014-0155-x

**Published:** 2014-09-17

**Authors:** Anne-Laure Dinel, Corinne Joffre, Pierre Trifilieff, Agnes Aubert, Aline Foury, Pascale Le Ruyet, Sophie Layé

**Affiliations:** Nutrition et Neurobiologie Intégrée, INRA UMR 1286, Bâtiment UFR Pharmacie, 2° tranche, 2° étage, Case courrier 34, Université Victor Ségalen, 146 rue Léo Saignat, 33076 Bordeaux, France; University of Bordeaux, Bordeaux, France; Lactalis, R&D, Retiers, F-35240 France

**Keywords:** lipopolysaccharide, cytokines, perinatal, anxiety, depression, cognition, neurogenesis, adolescence, adulthood

## Abstract

**Background:**

The postnatal period is a critical time window during which inflammatory events have significant and enduring effects on the brain, and as a consequence, induce alterations of emotional behavior and/or cognition later in life. However, the long-term effect of neonatal inflammation on behavior during adolescence, a sensitive period for the development of neurodevelopmental psychiatric disorders, has been little studied. In this study, we examined whether an early-life inflammatory challenge could alter emotional behaviors and spatial memory at adolescence and adulthood and whether stress axis activity, inflammatory response and neurogenesis were affected.

**Methods:**

Lipopolysaccharide (LPS, 100 μg/kg) was administered to mice on postnatal day (PND) 14 and cytokine expression was measured in the plasma and in brain structures 3 hours later. Anxiety-like and depressive-like behavior (measured in the novelty-suppressed feeding test and the forced swim test, respectively) and spatial memory (Y-maze test) were measured at adolescence (PND30) and adulthood (PND90). Hypothalamic-pituitary-adrenal (HPA) axis activity (plasma corticosterone and glucocorticoid receptors in the hippocampus and prefrontal cortex) was measured at adulthood. In addition, the impact of a novel adult LPS challenge (100 μ/kg) was measured on spatial memory (Y-maze test), neurogenesis (doublecortin-positive cell numbers in the hippocampus) and plasma cytokine expression.

**Results:**

First, we show in PND14 pups that a peripheral administration of LPS induced the expression of pro- and anti-inflammatory cytokines in the plasma and brain structures that were studied 3 hours after administration. Anxiety-like behavior was altered in adolescent, but not in adult, mice, whereas depressive-like behavior was spared at adolescence and increased at adulthood. This was accompanied by a decreased phosphorylation of the glucocorticoid receptor in the prefrontal cortex, with no effect on corticosterone levels. Second, neonatal LPS treatment had no effect on spatial memory in adolescence and adulthood. However, a second challenge of LPS in adulthood impaired spatial memory performance and neurogenesis and increased circulating levels of CCL2.

**Conclusions:**

Our study shows for the first time, in mice, that a peripheral LPS treatment at PND14 differentially alters emotional behaviors, but not spatial memory, at adolescence and adulthood. The behavioral effect of LPS at PND14 could be attributed to HPA axis deregulation and neurogenesis impairment.

## Introduction

According to the concept of perinatal programming of adult behavior [1], the postnatal period is a critical time-window during which an adverse event can have significant and enduring effects on the development of the central nervous system, and as a consequence, on behavior later in life. In this context, an inflammatory event during brain development has been shown to strongly increase the risk for the development of psychiatric disorders such as autism or schizophrenia [2,3].

In rodents, the early-life exposure to lipopolysaccharide (LPS), a Gram-negative bacteria endotoxin that induces inflammatory response, increases the risk of developing emotional behavior [4,5] as well as cognitive [6] alterations at adulthood. In neonate rodents, immune challenge has been reported to stimulate the production of proinflammatory cytokines, such as interleukin(IL)-1β, IL-6 and tumor necrosis alpha (TNFα), both at the periphery and in the brain [7], together with enhanced corticosterone levels [8]. These factors have been proposed to adversely impacts fetal brain development [9,10] such as hippocampal neurogenesis [11]. Studies in rodents have shown that postnatal day (PND)14 is a crucial period for brain network maturation. Microglial cells actively engulf synaptic material and play a major role in synaptic pruning during postnatal development in mice [12]. This synaptic pruning is particularly efficient at PND14, and alteration in microglia function induced by an inflammatory event at this period may contribute to synaptic abnormalities seen in some neurodevelopmental disorders [13]. Moreover at PND14, the neonatal stress axis is no longer in a hyporesponsive state [14], and pups exhibit an enhanced response of adrenocorticotropic hormone (ACTH) and corticosterone to LPS [15] that could also contribute to altered brain development.

The disruption of brain development linked to inflammatory factors exposure can exert a marked deficit in neuroimmune response and HPA axis activity at adulthood. In rats, exposure to LPS in early life increases corticotrophin-releasing hormone (CRH) mRNA levels in the hypothalamus; decreases glucocorticoid receptor (GRs) density in the hypothalamus, hippocampus and frontal cortex [16]; and increases HPA responsiveness to stress at adulthood [16,17]. Hypothalamic cyclooxygenase(COX)-2 expression induced by LPS administration in adulthood was attenuated in neonatally LPS-treated rats [18]. Some authors proposed that the long-lasting impact of an early-life immune challenge on the neuroimmune response at adulthood is strongly linked to the heightened HPA responsiveness [19,20]. Additionally, it has been shown that neonatal immune challenges impact memory and cognitive ability in adults [21-24]. Neonatal LPS in rats leads to memory deficits in adulthood only if unmasked by a subsequent LPS treatment 24 h before learning or immediately after learning [8,23,25-27].

Adolescence is a period of neurobehavioral shaping during which the hippocampus, amygdala and prefrontal cortex undergo maturation [28]. During this developmental period, synaptic pruning, as well as myelination, increases in order to refine functional connectivity [29]. This dynamic process strengthens network cohesion and contributes to developmental changes in patterns of brain activation [30]. As a consequence, neurobiological changes induced by neonatal inflammation can be particularly problematic at adolescence [29]. A recent work reveals that LPS administration during the pre- and postnatal periods alters behavior in adolescent rats [31]. Despite strong evidences that neonatal exposure to infection has a significant influence on adult behavioral outcomes [32], the period of sensitivity to early-life immune activation at PND14 and the short-term and long-term behavioral consequences remain relatively unexplored, particularly at adolescence. Overall, more research is needed to explore the longitudinal effect of PND14 neonatal LPS administration on emotional behavior and memory and the mechanisms through which an early-life immune challenge leaves a permanent change in the developing brain, on the inflammatory system, and on HPA axis activity in particular.

In the current study on mice, we evaluated whether an exposure to LPS in neonatal life (here PND14) 1) alters emotional behavior and spatial memory at adolescence and adulthood; 2) is associated with HPA axis dysfunctions at adulthood as manifested by alterations in corticosterone and glucocorticoid receptors (GR) expression in the brain; and 3) alters memory, neurogenesis and inflammatory markers in response to a second hit of LPS at adulthood.

## Materials and methods

### Animals

All experiments were conducted according to the INRA Quality Reference System and to relevant French (Directive 87/148, Ministère de l’Agriculture et de la Pêche) and European (Directive 86/609, November 24th 1986, European Community) legislation. They followed ethical protocols approved by the Région Aquitaine Veterinary Services (Direction Départementale de la Protection des Animaux, approval ID: A33-063-920) and by the ethics committee of animal experimentation of Bordeaux (seisin N° 50120112-A). Every effort was made to minimize suffering and the number of animals used.

Unmated adult male and female swiss mice (CD-1 mice) were obtained from Janvier (St Berthevin, France). They were housed in same sex pairs in polypropylene cages and maintained in a temperature- and humidity-controlled pathogen-free facility on a 12 h light-dark cycle with *ad libitum* access to food and water. Fifteen litters were used. At P4, litters were harmonized at 12 pups/litter with as many males as possible and a minimum of 3 females. All studies were limited to males. Male pups were injected at PND14 [33] with LPS (E.coli, serotype 0127:B8, Sigma Aldrich (Lyon, France), dissolved in sterile endotoxin-free isotonic saline, 100 μg/kg, intraperitoneally (i.p.)). Endotoxin-free isotonic saline was used as the vehicle control. Each litter was balanced in LPS and control pups.

**Figure 1:**
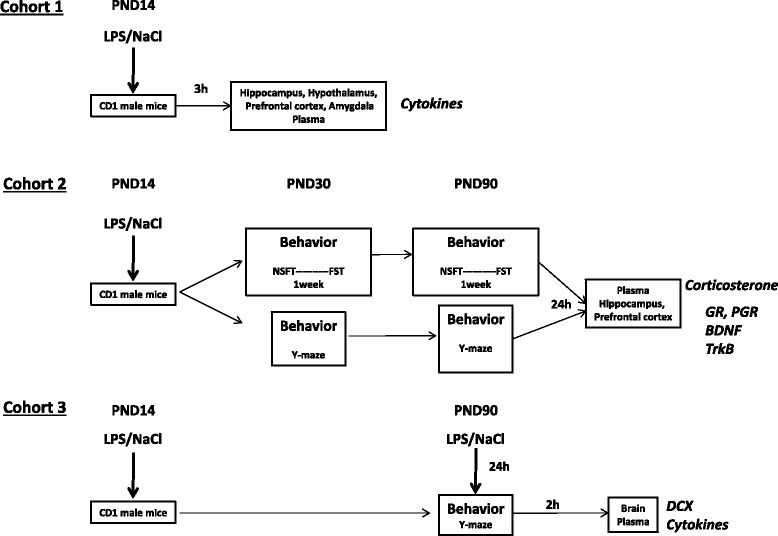
**Schematic timeline of the study design and experimental protocols.**

Three cohorts of mice were used (Figure 1). Cohort 1 was used to evaluate the effect of an LPS administration on brain cytokine production in the brain of PND14 mice pups. CD1 mice pups (PND14) (n = 20) received an injection of LPS and were sacrificed 3 h later. Plasma and brain structures (hippocampus, hypothalamus, prefrontal cortex and amygdala) were collected. Then, pro- and anti-inflammatory cytokine expression was measured by BioPlex in the plasma and by real time-PCR in brain structures.

Cohort 2 (n = 40) was used to evaluate the impact of neonatal LPS administration on emotional behavior and spatial memory at adolescence (PND30) and adulthood (PND90). In the first subgroup, PND30 mice were submitted to NFST and 1 week later to FST. Then, at PND90, mice were submitted to the same behavioral testing. In a second subgroup of mice, spatial memory was measured at adolescence (PND30) and at adulthood (PND90) using the Y-maze test in the same mice. All mice were sacrificed 24 h after the last behavioral test at PND90 to evaluate HPA axis activity and neurotrophin expression. After sacrifice, brain structures (hippocampus and prefrontal cortex) and plasma were collected. Expression of GR and its phosphorylated form, BDNF and its receptor trkB were measured by western blot from various brain structures and corticosterone levels were determined by radioimmunoassay (RIA) from the plasma.

Cohort 3 (n = 31) was used to evaluate the effect of a second hit of LPS administrated at adulthood on spatial memory, neurogenesis and cytokine production in the plasma. For this purpose, LPS-treated mice at PND14 and their controls were submitted to a LPS treatment (i.p., 100 μg/kg) at adulthood (PND90). Then, 24 h after administration of the second LPS treatment, spatial memory was assessed in the Y-maze test, and mice were sacrificed 2 h later. Brain and plasma were collected. Cytokines and chemokines were measured in the plasma (BioPlex) and doublecortin (DCX) immunohistochemistry was performed in the dentate gyrus of the hippocampus.

### Behavioral testing

#### Novelty-suppressed feeding test

The novelty-suppressed feeding test (NSFT) paradigm is a conflict test that elicits competing motivations between the drive to eat and the fear of venturing into the center of brightly lit arena. Latency to begin eating is used as an index of anxiety-like behavior because classical anxiolytic drugs as well as chronic treatment with selective serotonin reuptake inhibitor decrease this index [34]. Mice were food-deprived for 24 h prior to the test. Testing was performed in a 50 × 50 cm box covered with bedding and illuminated by a 70-watt lamp. The latency to eat pellets of food placed on the top of a piece of white filter paper in the center of the box was measured. Each mouse was tested individually for 5 min [34]. Two different arenas were used at P30 and at P90 to avoid potential interferences.

#### Forced swim test

The standardized forced swim test (FST) of depressive-like behavior was conducted as previously described [35,36]. Briefly, each mouse was placed individually in a cylinder (16 × 31 cm) containing warm water (25 ± 1°C) to avoid temperature-related stress response. Mice were tested during a 6-min period. Immobility time was assessed by measuring the time a mouse stopped swimming and moved only slowly to remain floating in the water, keeping its head above the water surface. Increased duration of immobility has been proposed to reflect a state of helplessness that is reduced by antidepressants.

#### Y-maze

Spontaneous spatial recognition in the Y-maze was used as a hippocampal-dependent test as previously described [36,37]. The apparatus consisted in a Y-shaped acrylic maze with three identical arms (34 × 8 × 14 cm). The floor was covered with corncob litter and was mixed between each trial in order to remove olfactory cues. Visual cues were placed on the walls of the testing room and kept constant during the whole test. Discrimination of novelty versus familiarity was based on the various environmental cues that the mouse can perceive from each arm of the Y-maze. In the first trial of the test (acquisition), one arm was closed with a door and mice were allowed to freely visit the two other arms for 5 min. After a 30-min intertrial interval (ITI), mice were again placed in the ‘start’ arm for the second trial (retrieval) and allowed free access to all three arms for 5 min. ‘Start’ and closed arms were randomly assigned to each mouse. Arm entries were defined as all four paws entering the arm. Preference for novelty was also measured using a short 2-min ITI between acquisition and retrieval in order to control for potential motivational disturbances [36,37]. Analyses were based on the time spent exploring the novel and the familiar arms during the 5 min of the second trial.

### Biochemical/histological measurements

#### Immunohistochemical detection of doublecortin -positive cells

After transcardiac perfusion with phosphate buffered saline (PBS, pH 7.4), followed by 4% paraformaldehyde (PFA), brains were removed, postfixed for 4 h in PFA, cryoprotected in 30% sucrose for 24 h, snap frozen in liquid nitrogen, and stored at -80°C before sectioning. Free-floating (30 μm) coronal sections containing the hippocampus (from -0.9 mm to -3.1 mm relative to bregma) were collected on a cryostat for immunohistochemistry.

Neurogenesis in the hippocampus was evaluated by determining the number of immature neurons in the dentate gyrus characterized by the endogenous marker doublecortin (DCX) (Santa Cruz Biotechnology, CA, USA), a cytoplasmic protein expressed transiently in newborn neurons only [38,39]. Briefly, 30-μm sections were incubated with rabbit anti-DCX antibody (1:800; Abcam, Cambridge, UK) followed by a biotinylated secondary antibody and streptavidin peroxidase complex, which was visualized by diaminobenzidine-nickel staining. The number of DCX positive cells was counted on three sections representing the same three levels of the dentate gyrus for all animals [39].

#### Measurement of neurotrophins, neurotrophin receptor and glucocorticoid receptors by western blot

Western blot analyses of the neurotrophic factor BDNF and its receptor TrkB and glucocorticoid receptor (GR) and its phosphorylated form (P-GR) were performed according to a previously published method [40]. Briefly, the hippocampus and prefrontal cortex were homogenized with a Precellys 24 system (Bertin Technologies, Aix en Provence, France) in lysis buffer (20 mM Tris, pH 7.5, 5 mM MgCl2, 1 mM DTT, 0.5 M EDTA,2 mM sodium orthovanadate (Na3VO4), and 1 mM NaF) containing protease inhibitor cocktail (Sigma, Saint-Louis, MO, USA). After centrifugation, protein concentration was determined using a BCA assay kit (Uptima, Montlucon, France). Equal amount of proteins (50 μg) were loaded onto SDS-PAGE gels (10% acrylamide) and transferred onto polyvinylidene difluoride (PVDF) membranes (Millipore, Billerico, MA, USA). Membranes were incubated overnight at 4°C with the following primary antibodies: anti-glucocorticoid receptor (GR) (M-20) (1:2000, Santa Cruz Biotechnology, Santa Cruz, CA, USA), anti-phospho-GR (Ser211) and anti-TrkB (80E3) (1:1000; Cell Signaling Technology, Boston, MA, USA), anti-BDNF (1:1000; Abcam, Paris, France) and anti-Actin (1:5000; Sigma, St-Louis, MO, USA). After being washed, membranes were incubated with peroxidase-conjugated secondary anti-rabbit antibody for 1 h (1:5000; Jackson ImmunoResearch laboratories, Westgrove, PA, USA). Between successive probing with antibodies, membranes were incubated for 15 min in stripping buffer (ReBlot Plus Strong Antibody Stripping Solution, Millipore) in order to remove the previous antibody. Staining was revealed with the ECL-Plus Western blotting detection system (Perkin Elmer, Forest City, CA, USA) or Lumina Forte Western HRP substrate (Millipore, Billerico, MA, USA). Chemiluminescence was captured by a Syngene detection system and quantified by Gene Tools software (Syngene, Cambridge, UK).

#### Assessment of corticosterone by radioimmunoassay

Total plasma corticosterone (CORT) was measured by an in-house radioimmunoassay (RIA, for details, see Ref. [41]). Briefly, after steroid extraction of plasma samples with absolute ethanol, total CORT was measured by competition between cold CORT and ^3^H-CORT by a specific anti-CORT antibody provided by H. Vaudry (University of Rouen, Rouen, France).

#### Assessment of cytokine and chemokine by BioPlex

Blood samples from mice were collected in EDTA-coated vials and centrifuged for 15 min at 3,000 g at 4°C, aliquoted and stored at -80°C. Milliplex map kits were used for all assays (Millipore, Molsheim, France) as previously described [36]. All samples were run in duplicate and were assayed for IL-1β, IL-6, TNF-α, IFN-γ, IL-10 at PND 14 and IL-1β, IL-6, TNF-α, IFN-γ, IL-10 and CCL2 at adulthood according to the manufacturer's instructions. Results were expressed in pg/ml. Minimum detectable concentration was 5.4, 1.1, 2.3, 1.1, 2.0 and 6.7 pg/ml, respectively.

#### Reverse transcription and real-time RT-PCR

Mice were sacrificed by cardiac puncture after isoflurane anesthesia and were transcardially perfused with PBS. Brain structures were quickly removed and frozen on dry ice. Two micrograms of total RNA were obtained from each brain area were reverse transcribed with SuperScript III reverse transcriptase (Invitrogen, Cergy-Pontoise, France). Quantitative PCR was then performed using the Applied Biosystems Assay-on- Demand Gene Expression Products protocol, as previously described [36,42]. Briefly, cDNAs for IL-6, IL-1β, TNF-α, IFNγ, IL-10 and a housekeeping gene (β2-microglobulin) were amplified by PCR using an oligonucleotide probe with a 5’ fluorescent reporter dye (6-FAM) and a 3’ quencher dye (NFQ). Fluorescence was measured using an AB 7500 Real-Time PCR system (Applied Biosystems, Foster city, CA), and final quantification was carried out using the comparative threshold (Ct) method as previously described [36,42]. For each experimental sample, difference between target gene and housekeeping gene Ct values (ΔCt) was used to normalize for differences in the amount of total nucleic acid added to each reaction and in the efficiency of the reverse transcription step. Values were then expressed as relative fold change (RFC) of the mean ΔCt value obtained for the group of control mice (calibrator ΔCt) by subtracting ΔCt for each experimental sample from the calibrator ΔCt ( = ΔΔCt) The amount of target gene (linear value) normalized to the housekeeping gene and relative to the calibrator was determined by 2 − ΔΔCt.

### Statistical analysis

All data are expressed as the mean ± SEM. Behavioral and biochemical data were statistically analyzed using comparisons between saline and LPS-treated mice at PND14 using an unpaired Student’s t-tests. A *P* value <0.05 was considered as significant. Data obtained after a second LPS stimulus were analyzed using a two-way ANOVA test with treatment at PND14 (saline versus LPS) and treatment at adulthood (saline versus LPS) as between factors, followed by Bonferroni post-hoc comparisons when appropriate.

## Results

### Lipopolysaccharide administration to postnatal day 14 mice induces the mRNA expression of pro- and anti-inflammatory cytokine in the brain

**Figure 2:**
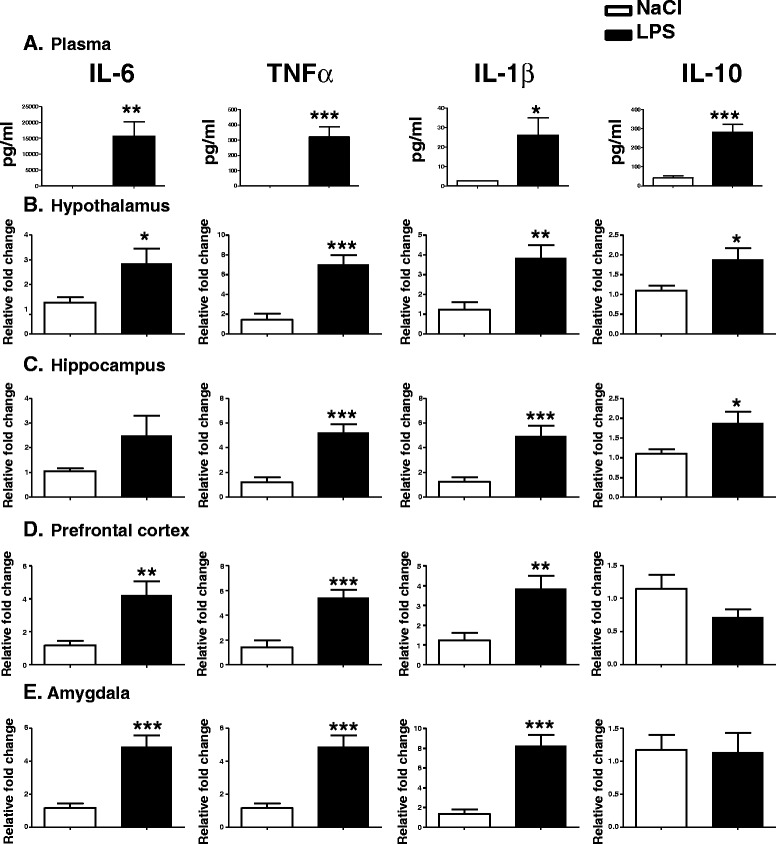
**Lipopolysaccharide (LPS) increases cytokines expression in plasma and brain structures of postnatal day 14 (PND14) mice.** Plasma concentration (pg/ml) of cytokines was measured by BioPlex 3 h after intraperitoneal (i.p.) administration of saline or LPS (100 μg/kg) in mice **(A)**. Cytokine mRNA expression was measured by real-time rt-PCR in the hypothalamus **(B)**, hippocampus **(C)**, prefrontal cortex **(D)** and amygdala **(E)**. Data are expressed as mean ± SEM, *P < .05, **P < .01, ***P < .001, for NaCl compared to LPS, n = 20.

We first investigated the effect of LPS injection (100 μg/kg) at PND14 on plasmatic cytokine production. As expected, LPS treatment induced a significant increase in plasma levels of IL-6 (t(17) = 3.290, *P* <0.01), TNF-α (t(19) = 4.492, *P* <0.001), IL-1β (t(18) = 2.627, *P* <0.05) and IL-10 (t(19) = 5.341, *P* <0.001) 3 hours later (Figure 2A).

We also measured cytokine mRNA expression 3 h postinjection in the hippocampus, hypothalamus, prefrontal cortex and amygdala (Figure 2B,C,D and E). LPS-induced IL-6 mRNA expression was significantly increased in the hypothalamus (t(19) = 2.229, *P* <0.05), prefrontal cortex (t(20) = 3.289, *P* <0.01) and amygdala (t(19) = 4.691, *P* <0.001). IL-1β and TNF-α expression was significantly increased in the hippocampus (t(20) = 3.890, *P* <0.001 and t(20) = 5.078, *P* <0.001, respectively), hypothalamus (t(18) = 2.338, *P* <0.05 and t(18) = 4.572, *P* <0.001, respectively), prefrontal cortex (t(19) = 3.130, *P* <0.01 and t(20) = 4.640, *P* <0.001, respectively) and amygdala (t(19) = 4.981, *P* <0.001 and t(19) = 4.691, *P* <0.001, respectively) 3 h after a LPS treatment. IL-10 mRNA expression was increased only in the hypothalamus and hippocampus (t(20) = 2.360, *P* <0.05 and t(20) = 2.3600, *P* <0.05 respectively) of LPS-treated mice, but there was no difference between both groups in the amygdala and the prefrontal cortex.

### Lipopolysaccharide treatment at postnatal day 14 alters emotional behavior of adolescent and adult mice

**Figure 3:**
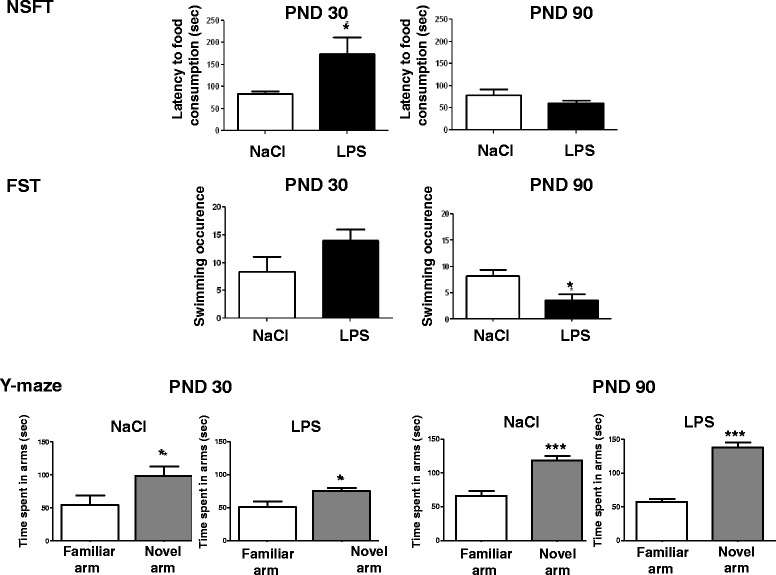
**Postnatal day 14 (PND14) neonatal lipopolysaccharide (LPS) differentially alters anxiety-like behavior, depressive-like behavior and spatial memory at adolescence (PND30) and adulthood (PND90).** Anxiety-like behavior was evaluated in the novelty-suppressed feeding test (NSFT) by measuring the latency (sec) of first food consumption in the center of the arena (n = 20). Depressive-like behavior was evaluated in the forced swim test (FST) by measuring the swimming occurrence (number) during 5 min (n = 20). Data are expressed as mean ± SEM. *P* <0.05. Spatial memory was evaluated in the Y-maze test by measuring the time spent exploring the novel and the familiar arms over a 5-min test and after a 30-min retention delay (n = 20). Data are expressed as mean ± SEM. *P <0.05, ***P <0.001, for familiar arm compared to novel arm.

Anxiety-like behavior of LPS-treated PND14 mice was measured in NSFT at adolescence (PND30) and adulthood (PND90). At PND30, the time for saline-treated mice to reach and start eating the food pellet in the center of the arena was significantly shorter as compared to the LPS-treated mice (*P* <0.05, t = 2.404) (Figure 3, top left panel). No significant difference in the latency to reach and eat the food pellet was measured in saline and LPS-treated mice at PND90 (Figure 3, top right panel). Even if neonatal LPS-treated mice slightly gained more weight than controls throughout life, no significant difference in weight loss was measured after fasting (data not shown), suggesting that the longer latency to eat the pellet is not related to a change in hunger in the LPS-treated animals.

At PND30, the measurement of swimming occurrences in the FST revealed no significant differences between groups (Figure 3, middle left panel). In contrast, LPS-treated PND14 mice presented a decreased number of swimming occurrences at PND90 compared to control mice (*P* <0.05, t = 2.749) (Figure 3, middle right panel).

No significant effect of the LPS treatment at PND14 was revealed on spatial memory performance measured at adolescence (PND30) or adulthood (PND90) in the Y-maze test with an ITI of 30 min (Figure 3, bottom panels left and right).

### Lipopolysaccharide treatment at postnatal day 14 alters glucocorticoid receptor phosphorylation in the prefrontal cortex of adult mice

**Figure 4:**
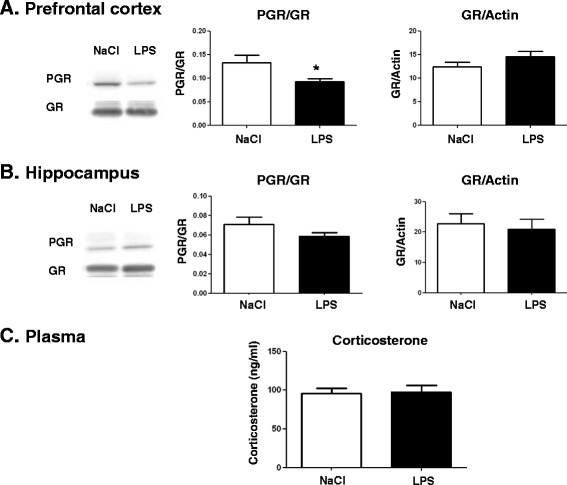
**Postnatal day 14 (PND14) neonatal lipopolysaccharide (LPS) decreases the phosphorylated glucocorticoid receptor/glucocorticoid receptor ratio in the prefrontal cortex at adulthood.** Representative immunoblots corresponding to the phosphorylated form of glucocorticoid receptor (PGR) and total glucocorticoid receptor (GR) in the prefrontal cortex **(A)** and the hippocampus **(B)** of adult mice receiving either NaCl or LPS at PND14 are presented. Immunoblots were quantified and the ratios for PGR/GR (left graph panels) or GR/actin were calculated (right graph panels). Data are expressed as mean ± SEM (n = 10/group). *P <0.05, for NaCl compared to LPS. Plasma corticosterone levels were measured by radioimmunoassay (RIA) in the plasma **(C)**. Data are expressed as mean ± SEM (n = 10/group). *P <0.05, for NaCl compared to LPS.

**Figure 5:**
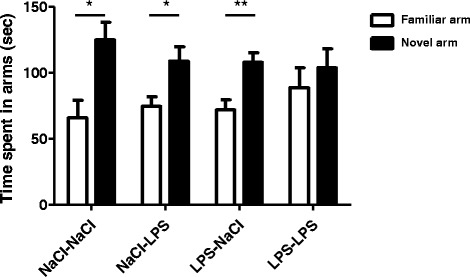
**A lipopolysaccharide (LPS) challenge at adulthood impairs spatial memory in postnatal day 14 (PND14) neonatal LPS mice.** Spatial memory performance was evaluated in the Y-maze test by measuring the time spent exploring the novel and the familiar arms. Measures were performed 24 h after intraperitoneal (i.p.) administration of saline or lipopolysaccharide (LPS, 100 μg/kg) in adult mice. They were assessed over a 5-min test and after a 30-min retention intertrial interval (ITI). Data are expressed as means ± SEM (n = 15/group). *P <0.05, **P <0.01, for familiar arm compared to novel arm.

Since emotional behavior is altered in adult mice treated with LPS at PND14, we next assessed the integrity of the stress system in brain structures involved in emotional behavior. Total GR and its phosphorylated form (PGR) were measured by western blot in the prefrontal cortex (Figure 4A) and the hippocampus (Figure 4B) of adult mice treated with LPS at PND14. No significant difference was detected for GR expression in the prefrontal cortex or the hippocampus. The PGR/GR ratio was significantly decreased in the prefrontal cortex (*P* <0.05, t = 2.629) (Figure 4A) but not in the hippocampus (Figure 4B), indicating a decrease of GR phosphorylation in the prefrontal cortex structure. Plasma corticosterone was not altered by PND14 LPS injection (Figure 5C). BDNF, TrkB and actin protein levels were measured in the hippocampus and the prefrontal cortex of adult mice by western blot. Expression of BDNF and its receptor TrkB was not impaired by LPS administered at PND14 (data not shown).

### Lipopolysaccharide treatment at adulthood impairs spatial memory performance in lipopolysaccharide-treated postnatal day 14 mice

We then evaluated the effect of a second LPS administration at adulthood on spatial memory (Figure 5). At adulthood, NaCl or LPS-treated mice at PND14 received an i.p. administration of NaCl (NaCl-NaCl and LPS-NaCl) or LPS (NaCl-LPS and LPS-LPS) and spatial memory was assessed using a Y-maze test 24 h later. Spatial memory differed across the treatments since only mice subjected to a double-hit of LPS spent the same time exploring the familiar arm and the novel arm (NaCl-NaCl, *P* <0.05, NaCl-LPS, *P* <0.05, LPS-NaCl, *P* <0.01, LPS-LPS, *P* = 0.166) (Figure 5). Total locomotion was not affected by the LPS treatment (data not shown).

### Lipopolysaccharide treatment at adulthood decreases the number of doublecortin-positive cells in the dentate gyrus of the hippocampus of lipopolysaccharide-treated postnatal day 14 mice

**Figure 6:**
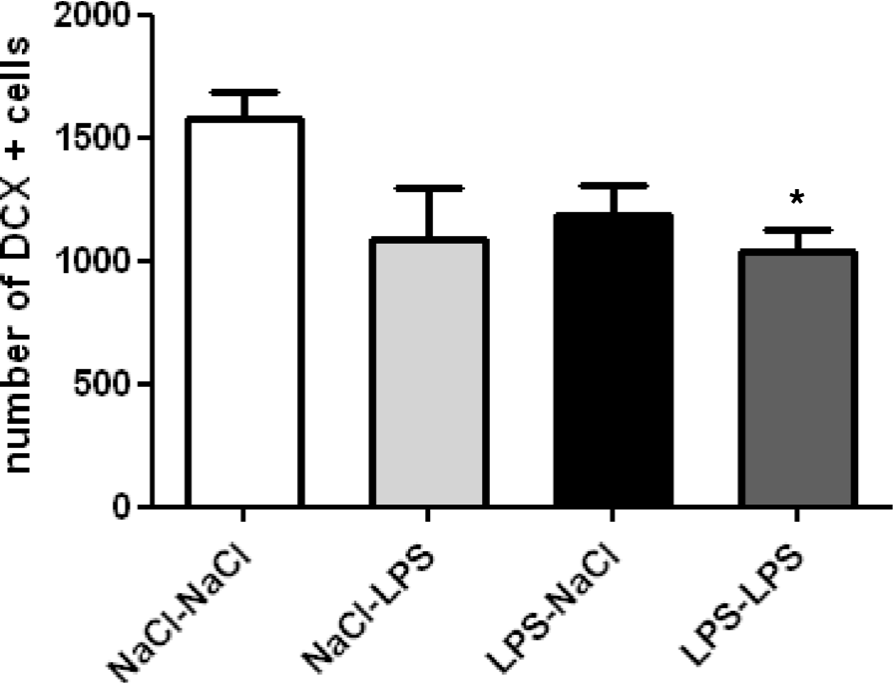
**A lipopolysaccharide (LPS) challenge at adulthood reduces the number of doublecortin (DCX)-positive cells in the dentate gyrus of the hippocampus of postnatal day 14 (PND14) neonatal LPS mice.** Neurogenesis was evaluated by quantifying the number of DCX-positive cells in the dentate gyrus of the hippocampus. Data are expressed as means ± SEM (n = 8/group). *P <0.05, for the NaCl-NaCl group compared to the LPS-LPS group.

Immature DCX-expressing neurons play an important role in spatial memory, particularly in learning [39,43]. We next assessed whether the memory impairment induced by the LPS-LPS treatment was accompanied by an alteration in the number of DCX-positive cells in the dentate gyrus. No significant effect of a single LPS administration either at PND14 or at adulthood was detected on the number of DCX-positive cells in the dentate gyrus of the hippocampus of adult mice. However, a second exposure to LPS at adulthood primed by an injection of LPS at PND14 resulted in a significant decrease in the number of DCX-positive cells (-25%) as compared to saline-treated animals (*P* <0.05) (Figure 6).

### Lipopolysaccharide treatment at adulthood increases CCL2 in the plasma of lipopolysaccharide-treated postnatal day 14 mice

In adult animals, hippocampal neurogenesis participates in hippocampal-dependent memory [44] and is influenced by proinflammatory cytokines and chemokines, such as CCL2 [45,46]. We then asked whether the priming effect of PND14 LPS administration on LPS injection in adulthood could be related to an alteration in the sensitivity of the immune system response. Cytokines (IL-6, IL-1β, TNF-α, IFN-γ and IL-10) and CCL2 (chemokine C-C motif ligand 2, also called MCP-1) were measured in the plasma 24 h after the second injection of LPS at adulthood.

**Figure 7:**
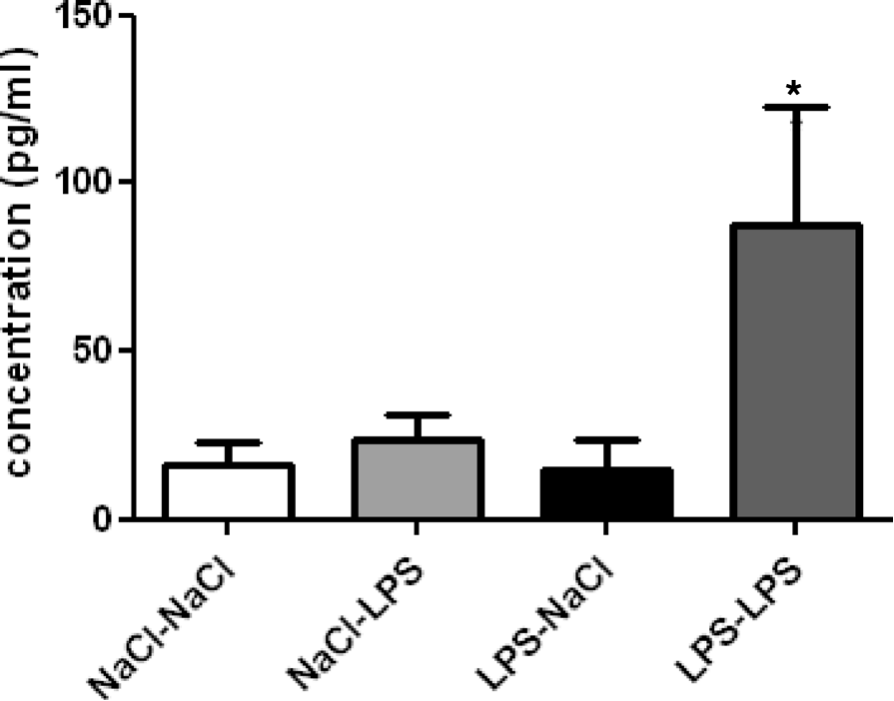
**A lipopolysaccharide (LPS) challenge at adulthood increases CCL2 in the plasma of postnatal day 14 (PND14) neonatal LPS mice.** CCL2 was measured in the plasma of adult mice by BioPlex. Data are expressed as means ± SEM (n = 7/group). *P = 0.05, for the NaCl-NaCl group compared to the LPS-LPS group.

No significant effect of LPS on plasmatic cytokine levels was revealed (data not shown). Of importance, CCL2 was significantly increased in the blood of mice treated with LPS at PND14 and at adulthood (LPS-LPS) compared to saline-treated mice (LPS-NaCl) (*P* = 0.05) (Figure 7).

## Discussion

The present study demonstrates that a peripheral LPS treatment at PND14, used to activate cytokine production in the brain, induces an alteration of emotional behaviors at adolescence (PND30) and adulthood (PND90). As a result, LPS-treated PND14 mice displayed altered anxiety-like behavior measured in the NSFT at adolescence, but not at adulthood. Of note, depressive-like behavior measured in the FST developed only at adulthood. This was accompanied by a decreased phosphorylation of the GR receptor in the prefrontal cortex, with no effect on circulating corticosterone levels. Second, PND14-LPS treatment had no effect on spatial memory measured in the Y-maze. However, a second administration of LPS at adulthood impairs spatial memory, DCX-positive cell numbers in the dentate gyrus and CCL2 circulating levels suggesting that LPS-treated PND14 mice were more vulnerable to LPS treatment at adulthood compared to naïve mice.

Taken together, our study shows for the first time that, in mice, a peripheral LPS treatment at PND14 differentially alters anxiety-like and depressive-like behavior at adolescence and adulthood. In adult mice, emotional behavior alteration is accompanied by a decreased phosphorylation of the glucocorticoid receptor in the prefrontal cortex. In addition, PND14,LPS treatment has no effect on spatial memory. However, a second administration of LPS at adulthood alters spatial memory, neurogenesis and peripheral chemokine CCL2 levels.

In the current study, we first measured the expression of pro- and anti-inflammatory cytokine expression in the plasma, the prefrontal cortex, the hippocampus, the hypothalamus and the amygdala of PND14 mice 3 h after LPS treatment. As previously shown, a single injection of LPS (100 μg/kg) in adult mice induced the expression of the cytokines in the plasma and brain structures [47,48]. Our results further revealed that at this dose, a single injection of LPS at PND14 activated the expression of both pro- (IL-6, TNF-α and IL-1β) and anti-inflammatory (IL-10) cytokine mRNA expression in several brain structures. However, the cytokine expression profile differed among the brain structures investigated. IL-1β, IL-6, TNF-α and IL-10 mRNA levels were significantly higher in the hypothalamus and the hippocampus. However, whereas IL-1β, IL-6 and TNF-α expression was induced in the amygdala and the prefrontal cortex, IL-10 mRNA expression was not. IL-10 is a potent anti-inflammatory cytokine [49]. In the brain, IL-1β and TNF-α expression is regulated by IL-10 [50]. In addition, IL-10 suppresses TNF-α-induced sickness behavior [51] and reverses the impairment in long-term potentiation (LTP) associated with increased IL-1β expression [52,53]. Decreased IL-10 production and activity in the brain has been previously shown to increase IL-1β expression [54-56]. It is then plausible that IL-10 induction acts as a negative feedback on pro-inflammatory cytokines IL-1β and TNF-α in the hypothalamus and the hippocampus, but not in the amygdala and the prefrontal cortex of PND14-LPS-treated mice.

**PND14-LPS administration has a significant impact on emotional behavior both at adolescence and adulthood.**

The latency to consume food placed in the center of the arena in the NSFT was used as a behavioral measure for anxiety in adolescent (PND30) or adult (PND90) mice.LPS-treated PND14 mice presented an increased latency in the NSFT at adolescence but not adulthood, indicating a higher level of anxiety-like behaviors in the PND14-LPS adolescent mice compared to the control. These differences could not be attributed to altered locomotor activity since the mice showed no difference in locomotion (data not shown). We also evaluated depressive-like behavior at both periods (PND30 and PND90), and we reported an increased occurrence of immobility in the FST only in adult LPS-treated PND14 mice, showing a higher level of depressive-like behaviors. We reported here for the first time a dichotomic pattern between anxiety-like and depressive-like symptoms induced by postnatal inflammation. Previous studies demonstrated an aversive effect of endotoxin exposure in early life on anxiety during adulthood [57] but not during adolescence [58] and on depressive-like behaviors [59]. But, these results were obtained with a neonatal inflammatory event occurring between PND3 and PND5, which could account for the discrepancy since it has been shown that the period of postnatal exposure to LPS impacts the perinatal programming of adult behavior. Indeed, Spencer *et al.* demonstrated that there is not a single ‘critical developmental window’ but that distinct aspects of adult physiology are affected by challenges at different developmental stages. One of these critical windows is after PND7 and before PND28, periods during which a single immune challenge is able to alter response to a second LPS challenge at adulthood [60]. PND14 has previously been shown to be a critical developmental period for the effect of an immune challenge, by reducing neuroimmune response to LPS in adulthood in rats as well as in mice [6,60,61]. These studies described a long-lasting altered innate immune response following a single low-dose immune challenge applied between PND7 and PND28. Galic *et al.* also demonstrated in rats that LPS injection at PND14 results in a greater seizure susceptibility to convulsants that depends on TNF-α, 6 to 8 weeks after postnatal LPS injection, indicating that a single LPS injection during a critical postnatal period causes a long-lasting increase in seizure susceptibility [62]. Interestingly, it has also been shown that a neonatal (PND7-8) LPS injection in the ventral hippocampus elicits persistent increases in IL-1 and IL-2 in the hippocampus, prefrontal cortex and nucleus accumbens that extend into adulthood [63].

**PND14-LPS administration induces a decrease of GR phosphorylation in the prefrontal cortex.**

Enhanced inflammatory state is associated with HPA-axis disturbances in some psychiatric disorders such as depression [64]. To understand the impact of postnatal LPS on depressive-like behaviors at adulthood, we investigated the glucocorticoid pathway in two structures implicated in mood and cognitive behaviors: the prefrontal cortex and the hippocampus. Even if PND-14 LPS injection did not impair the basal levels of corticosterone at adulthood, it induced a decrease in the phosphorylation of GR in the prefrontal cortex but no change in the hippocampus. Several studies demonstrated that certain subsets of depression could be due to glucocorticoid resistance associated with impairment of GR function [65,66]. The GR knockdown confined to the infralimbic prefrontal cortex caused acute stress hyper-responsiveness, sensitization of stress responses after chronic variable stress, and depression-like behavior [67]. Anacker *et al.* recently showed that the antidepressant-induced changes in neurogenesis are dependent on the GR [68]. Specifically, they showed that a selective serotonin reuptake inhibitor increases neuronal differentiation and promotes neuronal maturation of human hippocampal progenitor cells via a GR-dependent mechanism that is associated with an increase of GR phosphorylation. Cytokines and their downstream signaling molecules can influence the expression and activity of various factors that regulate local glucocorticoid bioavailability and GR function [69]. As demonstrated in the current study, the inflammatory response to postnatal LPS was structure-dependent, and this modulation of inflammation between prefrontal cortex and hippocampus could explain the difference observed in GR phosphorylation. Indeed studies on the effects of cytokines on GR function have consistently demonstrated that a variety of cytokines can inhibit GR signaling as reflected by decreased GR translocation in the nucleus and decreased activation of relevant GR-inducible enzymes or reporter gene constructs [70].

**A second hit of LPS at adulthood in PND14-LPS animals induces alterations in hippocampal-dependent memory and neurogenesis.**

It has been shown that the developing hippocampus is vulnerable to an early-life inflammation, since a single neonatal infection can affect cognitive processes such as learning and memory in adulthood [8]. In our context, a single PND14-LPS injection did not impact spatial working memory at adolescence and adulthood. But there are two potential mechanisms by which a neonatal inflammation might influence adult neural function and associated learning and memory [32]. Early life immune activation could permanently damage or disrupt the development of neural pathways important for learning and memory within the hippocampus, or alternatively, early-life immune activation could reprogram immune function, thereby negatively affecting how the adult immune system responds to a subsequent immune challenge via either prolonged or exaggerated pro-inflammatory cytokine production or decreased anti-inflammatory regulation. In this latter case, abnormal levels of cytokines and chemokines would indirectly impair the neural processes important for learning and memory. It appears to be difficult to define a sensitive period for the long-term cognitive effects of early-life inflammation [32]. In our study, an immune challenge at PND14 affects immune function and HPA axis modulation, sensitizing the system to a second immune challenge at adulthood. Adult mice were challenged with a second LPS injection at PND90. Twenty-four hours after this second challenge, animals displayed spatial working memory deficit.

This alteration of cognitive ability in animals receiving both juvenile and adult LPS injection was associated to a decrease in the number of DCX-positive cells in the dentate gyrus. In adult, LPS has been reported to diminish neurogenesis in the dentate gyrus of mammalian brain via the activation of microglial cells [71-73]. Here, we observed an increase in CCL2 chemokine levels in animals receiving both juvenile and adult LPS injection (*P* = 0.06), but no modulation of other peripheral cytokines (IL-6, IL-1β, TNF-α and IFN-γ). Increasing peripheral CCL2 chemokine levels *in vivo* in young mice decreased adult neurogenesis [74]. Many chemokines have a demonstrated impact on microglial migration and neural development of the healthy brain. CCL2 level is associated with cognitive decline, as demonstrated in aged mouse [74] and in Alzheimer patients [46]. In this study, we demonstrated that a double-hit challenge with LPS induced alterations of cognitive ability associated with a decrease in hippocampal neurogenesis and a modulation of HPA activity.

In conclusion, our data suggest that a PND14 immune challenge induces anxiety-like and depressive-like symptoms throughout life. This is accompanied by a change in GR activation in the prefrontal cortex, a structure involved in the regulation of mood and emotional behaviors. This postnatal inflammation seems to induce a ‘perinatal programming,’ in which the immune system could be approved. In this context, at adulthood, a second inflammatory event induced cognitive impairment associated with a decrease in hippocampus neurogenesis and a modulation of chemokine production. These findings may provide keys to better understand this crucial postnatal period.

## Abbreviations

ACTH: adrenocorticotropic hormone
BDNF: brain-derived neurotrophic factor
CCL2: chemokine C-C motif ligand 2
CRH: corticotrophin-releasing hormone
DCX: doublecortin
CORT: corticosterone
FST: forced swim test
GR: glucocorticoid receptor
HPA: hypothalamic-pituitary-adrenal
IL: interleukin
IFN: interferon
LPS: lipopolysaccharide
NFST: novelty suppressed feeding test
PBS: phosphate buffer saline
PND: postnatal day
RIA: radioimmunoassay
TNF: tumor necrosis factor
